# Maternal methyl donor supplementation regulates the effects of cafeteria diet on behavioral changes and nutritional status in male offspring

**DOI:** 10.29219/fnr.v67.9828

**Published:** 2023-10-27

**Authors:** Katya Herrera, Roger Maldonado-Ruiz, Alberto Camacho-Morales, Ana Laura de la Garza, Heriberto Castro

**Affiliations:** 1 Universidad Autonoma de Nuevo León, Facultad de Salud Pública y Nutrición, Centro de Investigación en Nutrición y Salud Pública. Monterrey, Nuevo León, México; 2 Universidad Autonoma de Nuevo Leon, Unidad de Neurometabolismo, Centro de Investigación y Desarrollo en Ciencias de la Salud. Monterrey, Nuevo León, México; 3 Universidad Autonoma de Nuevo Leon, Facultad de Medicina, Departamento de Bioquímica. Monterrey, Nuevo León, México; 4 Universidad Autonoma de Nuevo Leon, Unidad de Nutrición, Centro de Investigación y Desarrollo en Ciencias de la Salud. Monterrey, Nuevo León, México

**Keywords:** autism spectrum disorders, fetal programming, high-fat diet, social behavior, anxiety-like behavior, weight gain, food intakedate

## Abstract

**Background:**

Nutritional status and maternal feeding during the perinatal and postnatal periods can program the offspring to develop long-term health alterations. Epidemiologic studies have demonstrated an association between maternal obesity and intellectual disability/cognitive deficits like autism spectrum disorders (ASDs) in offspring. Experimental findings have consistently been indicating that maternal supplementation with methyl donors, attenuated the social alterations and repetitive behavior in offspring.

**Objective:**

This study aims to analyze the effect of maternal cafeteria diet and methyl donor-supplemented diets on social, anxiety-like, and repetitive behavior in male offspring, besides evaluating weight gain and food intake in both dams and male offspring.

**Design:**

C57BL/6 female mice were randomized into four dietary formulas: control Chow (CT), cafeteria (CAF), control + methyl donor (CT+M), and cafeteria + methyl donor (CAF+M) during the pre-gestational, gestational, and lactation period. Behavioral phenotyping in the offspring was performed by 2-month-old using Three-Chamber Test, Open Field Test, and Marble Burying Test.

**Results:**

We found that offspring prenatally exposed to CAF diet displayed less social interaction index when compared with subjects exposed to Chow diet (CT group). Notably, offspring exposed to CAF+M diet recovered social interaction when compared to the CAF group.

**Discussion:**

These findings suggest that maternal CAF diet is efficient in promoting reduced social interaction in murine models. In our study, we hypothesized that a maternal methyl donor supplementation could improve the behavioral alterations expected in maternal CAF diet offspring.

**Conclusions:**

The CAF diet also contributed to a social deficit and anxiety-like behavior in the offspring. On the other hand, a maternal methyl donor-supplemented CAF diet normalized the social interaction in the offspring although it led to an increase in anxiety-like behaviors. These findings suggest that a methyl donor supplementation could protect against aberrant social behavior probably targeting key genes related to neurotransmitter pathways.

## Popular scientific summary

- There are no studies that analyze the cafeteria and methyl donor diet in the context of sociability in offspring.
- The cafeteria diet also contributed to a social deficit and anxiety-like behavior in the offspring.
- A maternal methyl donor-supplemented cafeteria diet normalized the social interaction in the offspring although it led to an increase in anxiety-like behaviors.

Maternal obesity or overnutrition during pregnancy and lactation can lead to defects in the metabolic profile, and also neuropsychiatric disorders in the offspring, the literature indicates that these effects are generally found in animals, rodents, and primates (1–3). Maternal programing involves a new set of peripheral and central pathways including energy expenditure and inflammatory response, which potentially increase the susceptibility for neurodevelopmental disorders, such as autism spectrum disorder (ASD) in humans (4) and rodents (5). Several diet-induced obesity models, including the high-fat diet and the cafeteria (CAF) diet (6), have been confirmed to disturb the metabolic response to feeding and fasting in key metabolic organs in a murine model (7).

Several clinical studies have identified that maternal obesity leads to up to 1.39 % of ASD cases (8–10). ASD is a neurodevelopmental disorder characterized by persistent deficits in social communication and interaction as well as restricted, repetitive patterns of behaviors, interests, or activities such as stereotyped or repetitive motor movements. In addition, ASD subjects experience epilepsy, gastrointestinal disorders such as constipation, diarrhea, gastroesophageal reflux, abdominal pain, malabsorption, and dysbiosis (11). Also, anxiety-like behavior is one of the common comorbidities (12). Teixeira et al. demonstrated in rats that a maternal CAF diet can develop a social deficit in offspring. Eating patterns during pregnancy could play a role in regulating such behavior. Based on the above, methyl donors are key epigenetic mechanisms regulators that can be also a component of the diet.

The ‘epigenetic diet’ contains methyl group donors, including folic acid, betaine, choline, and cobalamin (13, 14), which can directly or indirectly act as methyl donors in DNA methylation (15) and regulate genome function during critical stages of development in mammals (16). Multiple forms of DNA methylation are recognised by methyl-CpG binding proteins (MeCPs), which play vital roles in chromatin-based transcriptional regulation, DNA repair, and replication (17). Accordingly, evidence for abnormal DNA methylation in ASD can be seen on multiple levels, from genetic mutations in epigenetic machinery to loci-specific and genome-wide changes in DNA methylation (18). About, activation of the maternal immune system could also lead to remodeling of some chromosomal regions and lead to changes in gene expression in the brain of the offspring (19). Dysregulation of micro-RNA could also lead to abnormal gene methylation and change in expression of an autism-related gene (20). These evidences confirm methylome signatures in subjects diagnosed with ASD.

Maternal diet has been probed to modulate methylome signatures and autism-like behavior in diverse models. Animal models of ASD are evaluated based on three criteria: psychological construct, face, and predictive validation. Psychological construct refers to the rationality of model creation and its ability to mimic etiological of the disorder. Face validation refers to the ability to approximate the physiological and behavioral phenotypic characteristics of people with the disorder. Also, predictive validation refers to the ability to determine the effectiveness of the intervention, taking as reference a clinical population that presents the disorder (21). Based on these murine models, reports documented that maternal supplementation with betaine attenuated the social alterations and repetitive behavior in offspring (22). Accordingly, maternal choline supplementation during mating, pregnancy, and lactation in female BTBR mice reduced the repetitive behaviors in offspring (23). It is believed that systemic methyl donors might reach the brain and modulate behavior. For instance, a methyl donor-supplemented diet increased the concentration of folate intermediates in the prefrontal cortex (PFC), which is blunted in male, but not female offspring prenatally exposed to high-fat diet, confirming a sex-dependent effect supplementation (24). This evidence supports the potential role of the methyl donor supplementation on social behavior and nutritional status. Overall, we hypothesized that intake of methyl donors during pre-gestation, gestation, and lactation can improve the harmful effects of a CAF diet on social behavior in offspring.

This study aims to analyze the effect of maternal CAF diet and methyl donor-supplemented diets on social, anxiety-like, and repetitive behavior in male offspring, besides evaluating weight gain and food intake in both dams and male offspring.

## Materials and methods

### Diets

Table 1 shows the types of experimental diets used in this study. For the dams, the following diets were used: (1) standard chow diet (CT) formula that contained 3.35 kcal/g (Rodent Lab Chow diet 5001; LabDiet, St. Louis, MO, USA). (2) CAF (CAF) diet (3.72 kcal/g) that consisted of a mix of liquid chocolate, fried potatoes, bacon, biscuits, standard chow diet, and pork pate based on 1:1:1:1:1:2 ratio, respectively. CT and CAF diets supplemented with methyl donors (Sigma-Aldrich, St. Louis, MO, United States) consisted of (3) CT (CT + M) and (4) CAF formula (CAF + M) enriched with betaine (5 g/kg of diet), choline (5.37 g/kg of diet), folic acid (5.5 mg/kg of diet), and vitamin B_12_ (0.5 mg/kg of diet) (25–28).

**Table 1:** Composition of maternal diets during pre-gestation, gestation, and lactation

Diet	Standard^a^	Cafeteria^b^
Kcal/kg	3,350	3,720
Carbohydrates (kcal)	1,909	1,450
Protein (kcal)	1,005	4,466
Lipids (kcal)	4,355	1,822
Sodium (mg)	290	513.53
Folic acid (mg)	5.5	5.5

Kcal = kilocalorie, kg = kilogram, mg = milligram.

a Rodent Lab Chow diet 5001; LabDiet, St. Louis, MO, USA;

b Cruz-Carrillo, et al., 2020.

### Animals and experimental design

**Fig. 1:**
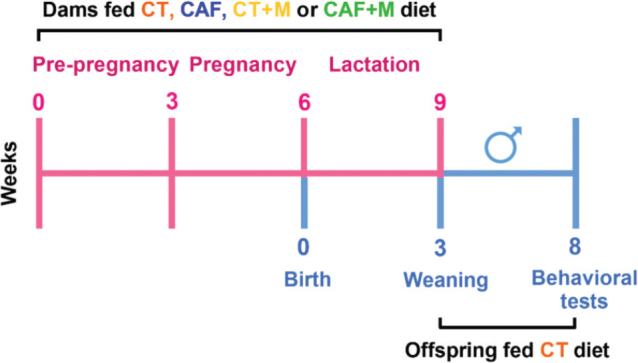
Maternal programming experimental design. Female C57BL/6 mice fed different diets during the pre-gestational, gestational and lactation period. At weaning, male offspring were fed only control diet until week 8 of age where behavioral tests were performed. CT = Control; CAF = Cafeteria; CT+M = Control + methyl donors; CAF+M = Cafeteria + methyl donors.


Virgin females C57BL/6 mice (Scientific, Technological, and Commercial Services S.A. de C.V., Monterrey, Mexico), housed in polypropylene boxes in an environment of 21–22°C with 12-h light/dark cycles and with free access to food and water. The animals were randomly distributed in four groups: standard chow diet (CT, *n* = 3), CAF diet (CAF, *n* = 5), CT + methyl donor supplemented diet (CT + M, *n* = 5), and CAF + methyl donor supplemented diet (CAF + M, *n* = 3). The animals were exposed to this diet for 9 weeks, including pre-gestation (before and during mating), gestation, and lactation. Mice were mated to similarly age and strain males. Male offspring were culled to 10 pups per dietary group and housed in cages after weaning at lactation-day 21 and then were exposed to a control diet until 8 weeks of age (Fig. 1). By the age of 2 months, male offspring were assigned to three behavioral tests, as detailed below. Weight gain was registered weekly. Similarly, food intake was measured weekly by weighing the initial amount of chow diet offered to each cage and what was left after 1 week, and energy intake was also determined. Energy efficiency (EE) was calculated to estimate the efficiency of the diet to promote weight gain expressed as g/kcal (29) with the following equation:

$$\mathrm{EE}=\frac{\mathrm{Weight}\;\mathrm{gain}\;\left(g\right)\;\mathrm{in}\;a\;\mathrm{determinated}\;\mathrm{time}}{\mathrm{Kcal}\;\mathrm{consumed}\;\mathrm{in}\;a\;\mathrm{determinated}\;\mathrm{time}}$$

Animals were handled according to the NIH guide for the care and use of laboratory animals (NIH Publications No. 80–23, revised in 1996), with approval of the local Animal Care Committee of the Facultad de Salud Pública y Nutrición with the identifier 19-FaSPyN-SA-17.TP. The procedures were carried out according to the Institutional Biosafety Committee of the Center for Research and Development in Health Sciences of the Universidad Autonoma de Nuevo León.

### Behavioral phenotyping

#### Three-Chamber Test

Sociability was evaluated by a three-chamber test. The apparatus was an acrylic box divided into three chambers. We quantified the time expended on left or right chamber (unfamiliar mouse or plastic square figure). Approach behavior was defined as interaction time including sniffing or approaching the target (unfamiliar mouse or plastic square figure) in each chamber (within 2 cm). The social and non-social parameters were analyzed by two observers (30).

#### Open Field Test

Mice were tested for general exploratory locomotion and anxiety-like behavior. The setup consisted of a rectangular acrylic arena (50 × 50 cm) surrounded by walls (38 cm). The mouse was placed in the center of the arena to freely explore during 10 min. Total traveled distance, duration in central and peripheral zones, activity, and freezing % of the time, rearing, and thigmotaxis duration were video recorded and analyzed using OMNIALVA, Inc., software (31).

#### Marble Burying Test

The mouse was placed in a cage with 5 cm fresh bedding and allowed 5 min for habituation. Then 16 green or blue glass marbles were placed into the cage while the mouse was temporarily removed from the cage. After that, the mouse was returned to the same cage for the 30-min test. The number of marbles buried was counted by two observers. A marble covered more than 70% of its diameter with bedding was considered to be buried (32).

### Statistical analysis

Data are presented as mean ± standard error of mean (SEM). Body weight and food intake were analyzed with a repeated-measures ANOVA with time (T), diet (D), and time x diet (TxD) as factors. Total weight gain, energetic intake, and EE were analyzed with a one-way ANOVA. Two-way ANOVA was performed to analyze the effect of maternal diet (D) × methyl donor supplementation (S) or maternal diet × methyl donor supplementation (DxS) on the behavior of the offspring. Post hoc analysis was performed with a Tukey’s test. A value of *P* < 0.05 (*P < 0.05; ** *P* < 0.01; *** *P* < 0.001) was considered as statistically significant between differences among the groups. SPSS v25 and GraphPad v8 were used for this study.

## Results

### Diet-related changes in weight gain and intake in dams

**Fig. 2:**
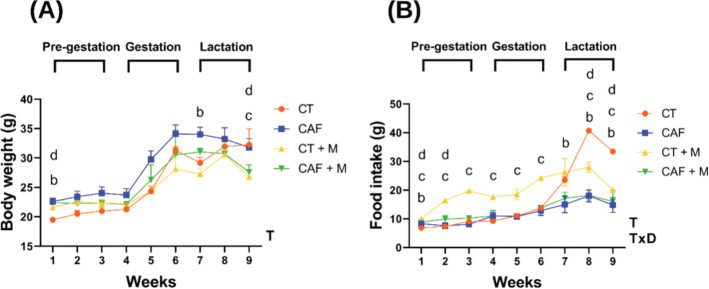
Body weight (A) and food intake (B) in dams. Control (CT, *n* = 3), Cafeteria (CAF, *n* = 5), Control + methyl donors (CT + M, *n* = 5) and CAF+M = Cafeteria + methyl donors (CAF + M, *n* = 3). Data is presented as mean ± SEM (*n* = ≥3 animals per group). Repeated-measures ANOVA was performed to analyze the effect of Time (T), Diet (D), or interactive effect of Time x Diet (TxD). Difference between groups was assessed by ^a^Tukey’s post hoc test. ^b^*P* < 0.05 (CT vs. CAF); ^c^*P* < 0.05 (CT vs. CT + M); ^d^*P* < 0.05 (CT vs. CAF + M).

During the pre-gestational period CAF, and CAF+M dams weighed significantly more at week 1 than the CT dams (*P* < 0.05). Baseline data indicated a similar behavior concerning weight (Table 2). All dams gained weight during the pregnancy, but there was no significant difference between dietary groups. During the lactation period, CAF dams displayed significantly higher body weight at week 7 (*P* < 0.05). Moreover, methyl donors-supplemented groups CT+M and CAF+M weighted significantly less at week 9 than the CT group (*P* < 0.05) (Fig. 2A). Regarding food intake, CAF dams presented a significantly higher intake during pre-gestation at week 1 (*P* < 0.001), CT+M dams at week 1 (*P* < 0.001), 2 (*P* < 0.001), and 3 (*P* < 0.001), as well as CAF+M dams at week 1 (*P* < 0.001) and 2 (*P* < 0.05) compared to the CT dams at this period. During gestation, CT+M dams showed significantly higher intake at weeks 4, 5 and 6 (*P* < 0.001) compared to the CT group. There was no significant difference between the other dietary groups. At lactation, all dietary groups displayed an opposite trend regarding eating behavior compared to the CT group. At week 7, CAF dams showed significantly less intake compared to CT dams (*P* < 0.05). Furthermore, all dams showed significantly less food intake at weeks 8 (*P* < 0.001) and 9 (*P* < 0.001) compared to the CT group (Fig. 2B). Regarding weight gain, both the supplemented groups (CT+M & CAF+M) gained significantly less weight compared to the CT group. Both control diet groups, supplemented or not (CT+M & CT) consumed significantly more total calories (605.00 ± 9.7 and 518.79 ± 4.2, respectively) compared to the other groups. The control group and the CAF group had a significantly higher EE (0.02 ± 0.002 and 0.02 ± 0.003) compared to the other groups (Table 3).

**Table 2:** Baseline data on body weight and food intake in dams

Maternal diet	Body weight (g)	Food intake (g)
Control	19.14 ± 0.3	6.30 ± 0.2
Cafeteria	22.55 ± 0.6*	8.66 ± 0.1***
Control + Methyl donors	21.53 ± 0.4	10.00 ± 0.0***
Cafeteria + Methyl donors	22.48 ± 0.8*	10.00 ± 0.0***

Difference between groups was analyzed by a one-way ANOVA and a Tukey´s post hoc test.

* *P* < 0.05 (vs CT);

*** *P* < 0.05 (vs. CT).

Groups: (CT, *n* = 3), (CAF, *n* = 5), (CT + M, *n* = 5), (CAF + M, *n* = 3).

**Table 3:** Nutritional status in dams from pre-gestation period to lactation

Maternal diet	Total weight gain (g)	Total energetic intake (kcal)	Energy efficiency (g/kcal)
Control	14.33 ± 1.4	518.79 ± 4.2	0.02 ± 0.002
Cafeteria	9.20 ± 1.6	395.87 ± 5.8^a, c^	0.02 ± 0.003^c^
Control + Methyl donors	5.19 ± 0.1^a^	605.00 ± 9.7^a, b, d^	0.00 ± 0.000^a, b^
Cafeteria + Methyl donors	5.19 ± 1.4^a^	431.79 ± 10.3^a, c^	0.01 ± 0.003^a^

Difference between groups was analyzed by a one-way ANOVA and a Tukey´s post hoc test.

a *P* < 0.05 (vs. CT),

b *P* < 0.05 (vs. CAF),

c *P* < 0.05 (vs. CT + M),

d *P* < 0.05 (vs. CAF + M).

Groups: (CT, *n* = 3), (CAF, *n* = 5), (CT + M, *n* = 5), (CAF + M, *n* = 3).

### Maternal diet programming effect on weight gain, food intake and EE in male offspring

**Fig. 3:**
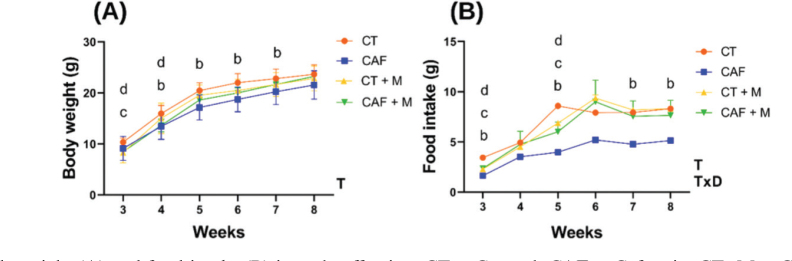
Body weight (A) and food intake (B) in male offspring. CT = Control, CAF = Cafeteria, CT+M = Control + methyl donors and CAF+M = Cafeteria + methyl donors. Data is presented as mean ± SEM (*n* = 10/group). Repeated-measures ANOVA was performed to analyze the effect of Time (T), Maternal diet (D), or interactive effect of Time x Maternal Diet (TxD). Difference between groups was assessed by ^a^Tukey’s post hoc test. ^b^*P* < 0.05 (CT vs. CAF); ^c^*P* < 0.05 (CT vs CT + M); ^d^*P* < 0.05 (CT vs. CAF + M).

The CAF offspring showed significantly lower body weight than the CT offspring at weeks 4, 6, and 7 (Fig. 3A). Regarding food intake, the CAF group presented significantly less intake at weeks 3, 5, 7, and 8 than the CT group (Fig. 3B). Even though the CAF offspring consumed significant fewer total calories (81.32 ± 1.0) compared to the other groups (*P* < 0.05), these animals had a significant higher EE compared to the CT and CT+M offspring (Table 4).

**Table 4:** Nutritional status in male offspring from weaning to early adulthood

Maternal diet	Total weight gain (g)	Total energetic intake (kcal)	Energy efficiency (g/kcal)
Control (CT)	13.30 ± 0.3	137.84 ± 0.6	0.09 ± 0.00
Cafeteria (CAF)	12.42 ± 0.6^c, d^	81.32 ± 1.0^a, c, d^	0.15 ± 0.01^a, c^
Control + Methyl donors (CT + M)	14.54 ± 0.5^b^	132.58 ± 4.3^b^	0.11 ± 0.01^b, d^
Cafeteria + Methyl donors (CAF + M)	14.76 ± 0.3^b^	125.15 ± 24.7^b^	0.15 ± 0.02^a, c^

Difference between groups was analyzed by a one-way ANOVA and a Tukey´s post hoc test (*n*= 10/group).

a *P* < 0.05 (vs. CT),

b *P* < 0.05 (vs. CAF),

c *P* < 0.05 (vs. CT + M),

d *P* < 0.05 (vs. CAF + M).

### Maternal diet influence on behavioral phenotyping in male offspring

**Fig. 4:**
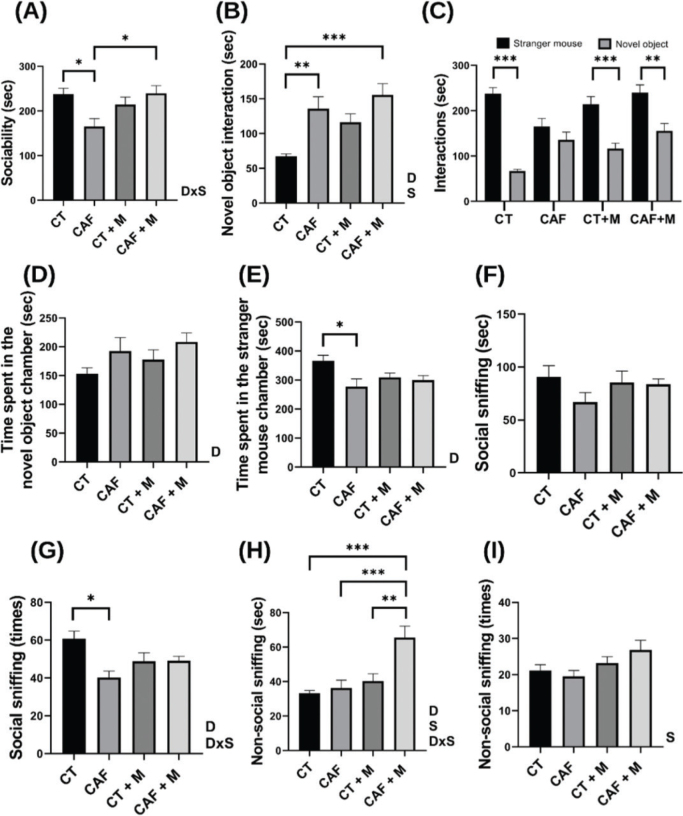
3-Chamber test results in male offspring. Sociability duration between experimental groups (A), comparation of novel object interaction duration between experimental groups (B), individual comparation of social and non-social interactions duration (C), time spent in the stranger mouse chamber (D), time spent in the novel object chamber (E), duration of social sniffing (F), times of social sniffing (G), non-social sniffing duration (H) & times of non-social sniffing (I) (*n* = 10/group). CT = Control, CAF = Cafeteria, CT+M = Control + methyl donors & CAF+M = Cafeteria + methyl donors. Two-way ANOVA was performed to analyze the effect of Maternal Diet (D), Supplementation of methyl donors (S), or Maternal Diet x Supplementation (DxS). Difference between groups was assessed by a Tukey´s post hoc test. *P < 0.05; ** *P* 0.01; *** *P* < 0.001.

In the 3-chamber test, the CAF offspring displayed significantly less time (sec) interacting with the stranger mouse (*P* < 0.05) (Fig. 4A), and higher interaction with the novel object or non-social interaction compared to the CT offspring (*P* < 0.01), also CAF+M offspring showed significantly higher time interacting with the novel object compared to the CT offspring (*P* < 0.001) showing that a maternal CAF diet, supplemented with methyl donors or not, can promote an alteration in this parameter (Fig. 4B). When comparing these two interactions (social and non-social), all the experimental groups presented a significant preference for interacting with the stranger mouse (*P* < 0.001) except for the CAF group (Fig. 4C). The CAF group displayed a significantly smaller number of total social sniffs compared to the CT group (*P* < 0.05) (Fig. 4G). The CAF+M group presented spent a greater time sniffing the novel object compared to all the experimental groups (*P* < 0.001) (Fig. 4H).

**Fig. 5:**
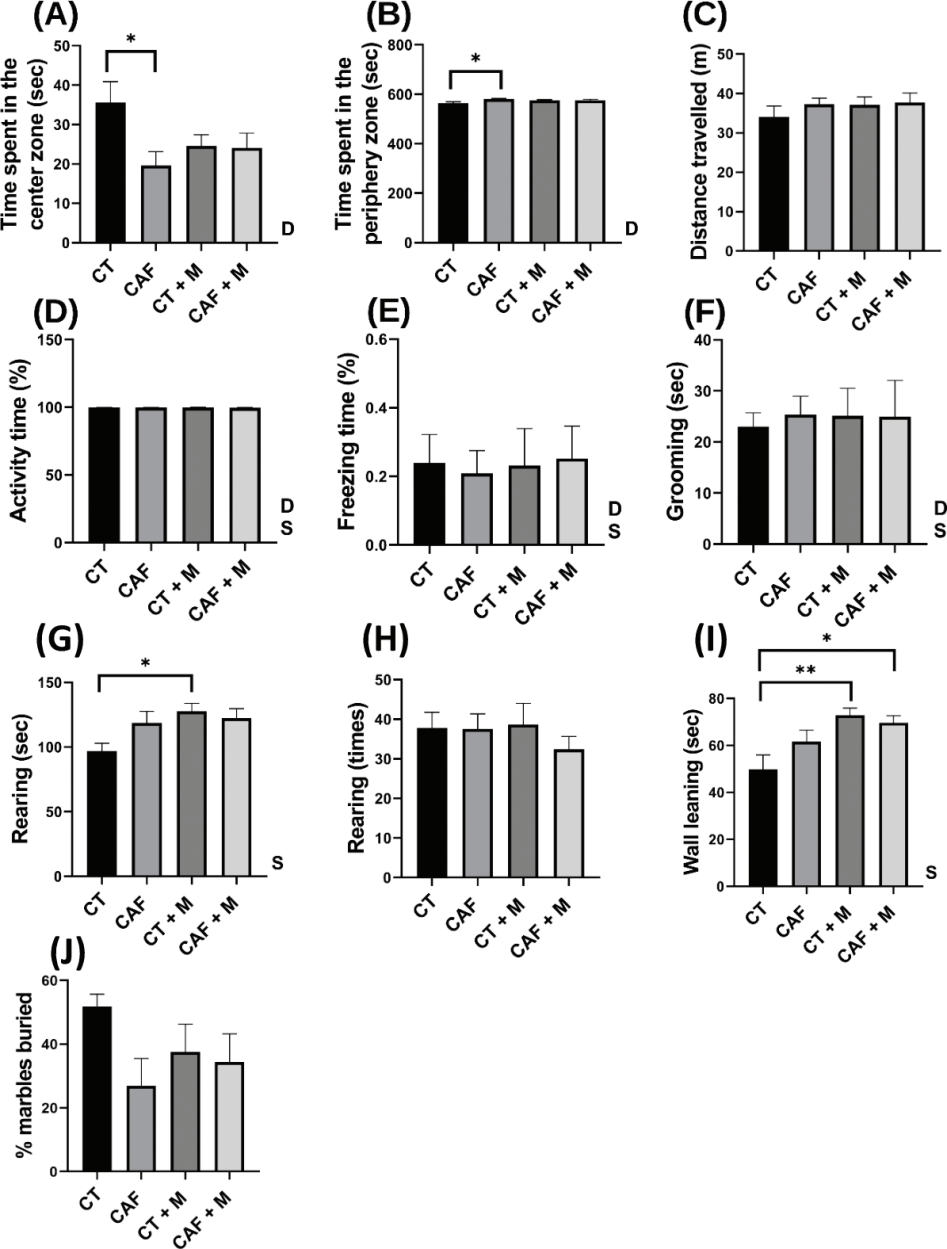
Open field and marble burying tests result in male offspring. Time spent in center zone (A), time spent in periphery zone (B), distance travelled (C), activity time (%) (D), freezing time (%) (E), grooming (F), duration of rearing (G), times of rearing (H), wall leaning (I), and percentage of marbles buried (J) (*n* = 10/group). CT = Control, CAF = Cafeteria, CT+M = Control + methyl donors and CAF+M = Cafeteria + methyl donors. Two-way ANOVA was performed to analyze the effect of Maternal Diet (D), Supplementation of methyl donors (S), or Maternal Diet x Supplementation (DxS). Difference between groups was assessed by a Tukey´s post hoc test. *P < 0.05; **P < 0.01; ***P < 0.001.

**Fig. 6:**
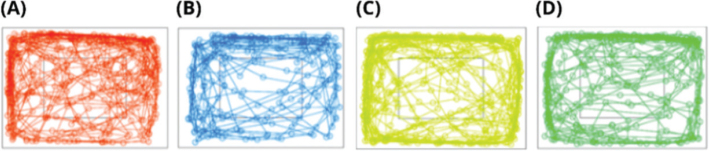
Traces of animal track paths across the periphery and center zone in the open field test in male offspring (*n* = 10/group): control (A), cafeteria (B), control + methyl donors (C) and cafeteria + methyl donors (D).

In the open field test (Figs. 5 and 6), the CAF group displayed significantly less time (sec) in the center zone (Fig. 5A) and greater time in the periphery zone compared to the CT offspring (*P* < 0.05) indicating an anxiety-like behavior (Fig. 5B & 6). There were no significant differences between groups in the parameters of distance traveled (m) (Fig. 5C), activity time (%) (Fig. 5D), freezing time (%) (Fig. 5E), and duration of grooming behavior (sec) (Fig. 5F). Regarding the anxiety-like related behaviors, the CT+M offspring showed a significantly greater duration (sec) of rearing compared to the CT offspring (*P* < 0.05) (Fig. 5G). The CT+M and CAF+M presented a significantly greater time spent leaning on the wall than the CT offspring (Fig. 5I). There were no significant differences between groups in the % of marbles buried that indicate repetitive behavior (Fig. 5J).

## Discussion

The high prevalence of maternal obesity and the consumption of a CAF diet contribute to several health complications in both the mother and offspring (33). Here, the CAF diet generates a greater gain in body weight; however, this effect is modified when supplemented with methyl donors, inducing weight loss, especially at the end of the lactation period. It is important to remark that the control group supplemented with methyl donors also presented a reduction in weight gain, which could suggest that this effect occurs from supplementation, regardless of the type of diet. On the other hand, it should also be considered that the supplemented mothers had a lower dietary intake during the lactation period, which could be contributing to the lower weight gain in this stage. The present study included supplementation based on methyl donors, including betaine. Animal studies have shown the beneficial effect of betaine supplementation on body composition, while the data from human studies are controversial and inconsistent. In this regard, oral betaine supplementation has been shown to reduce body weight and BMI compared to placebo-treated participants (34).

The results of the present study showed that a maternal CAF diet supplemented with methyl donors does not reduce weight gain in the offspring at the end of the study compared to the control group. Concerning the role of methyl donor supplementation, several authors have described the opposite effects of folic acid supplementation on weight (35). Cho et al. found that a high maternal folic acid supplementation promoted a greater weight and food intake in male offspring compared to the recommended folic acid supplementation group. They also found hypomethylation of 5-HTR2A and the pro-opiomelanocortin (POMC) promoter occurred with the high folate pup diet. POMC-specific methylation was positively associated with glucose response to a glucose load (36).Several lines of evidence support a role for serotonin (5-hydroxytryptamine [5-HT]) signaling in the regulation of eating behavior and long-term body weight (37). The experimental modulation of multiple serotonin receptor subtypes has been shown to affect food intake and/or body weight regulation in animal models. The serotonin system spans across the central and peripheral nervous system, and the central and peripheral components have opposing effects on energy homeostasis (38). Overall, central serotonergic signaling is anorexigenic, and it increases energy expenditure via the stimulation of thermogenesis in brown adipose tissue (39). Moreover, POMC neurons in the arcuate nucleus of the hypothalamus (ARC) play an essential role in the control of food intake and energy expenditure (40). In the melanocortin pathway to regulate feeding, hypothalamic POMC neurons receive inhibitory signals from cholinergic neurons localized to the dorsomedial hypothalamus (DMH) that project to the arcuate nucleus (41), as well as from excitatory glutamatergic signals from steroidogenic factor (SF-1)-expressing neurons localized to the ventromedial hypothalamus (42).

On the other hand, maternal CAF diet offspring had less weight and food intake compared to the control group; in this regard, Vithayatil et al. reported that offspring from CAF diet-fed dams were lighter at weaning and that this pattern was maintained at 6 weeks of age even when the pups were fed a chow diet compared to the offspring from control chow diet-fed dams. They suggest that this diet has nutritional deficiencies that affect fetal growth. This diet high in fat tends to be low in protein which is a fundamental nutrient for lowering the risk of restriction in intrauterine growth. A CAF diet also contains low-key micronutrients that are also needed for fetal growth and neurodevelopment (43). However, offspring from maternal CAF diet supplemented or not, had greater EE, showing that this diet can exert an obesogenic phenotype in later life (29).

Regarding social behavior, we found that a maternal CAF diet can program a social deficit in offspring. Teixeira et al. reported that offspring from dams fed CAF diet during lactation period presented fewer social interactions in comparison to the control group (13). Buffington et al. reported that a maternal high-fat diet (MHFD) can promote social alterations in offspring. They attribute this effect to the gut-microbiota-brain axis due to the detection of intestinal dysbiosis in these animals, to lower oxytocin levels in the paraventricular nuclei of the hypothalamus, and impairing of the mesolimbic dopamine reward system (44). These findings suggest that maternal CAF diet is efficient in promoting a reduced social interaction in murine models. High-fat meals like CAF diet can stimulate innate immune cells and lead to a transient postprandial inflammatory response, altering our immune system and subsequently our inflammatory status (45). Cell oxidative stress is another event that is closely linked to tissue inflammation. Nutritional, or dietary, oxidative stress denotes a disturbance in the redox state resulting from excess oxidative load or inadequate nutrient supply, favouring pro-oxidant reactions (46). Prenatal programming by exposure to high-energy diets and their effects on the offspring behavior seems to be primed at very early stages of development. Reports confirm that mothers provide an innate priming stimulus showing interleukin (IL)-6 accumulation in plasma after ingesting a high-fat diet feeding (47). Notably, higher maternal IL-6 concentration during pregnancy was associated with defective brain connectivity and cognitive performance in the newborn at 2 years of age (48, 49). Finally, peripheral administration of IL-6 could induce neurovascular remodeling leading to increased permeability of the blood–brain barrier and defective behavior in mice (50). This evidence supports a causal effect of IL-6 accumulation in dams exposed to high-energy diets during pregnancy affecting the establishment of brain circuits establishment at the fetus (51). In our study, we hypothesized that a maternal methyl donor supplementation could improve the behavioral alterations expected in maternal CAF diet offspring. There are no studies that analyze these two different diets in the context of sociability in offspring. Orenbuch et al. reported that a prenatal methyl donor diet (folic acid, betaine, and choline) in dams *Mthfr* ± lead to a higher number of social behaviors in offspring compared to those from the group of control diet in dams from the same genotype (52). Zhang et al. showed that a postnatal administration of folic acid in BTBR offspring promoted a greater duration of social interactions compared to the control group. They attribute this effect to the presence of an attenuation in levels of oxidative stress and inflammation markers such as interleukin-1β (IL-1β), Iba-1, IL-18, tumor necrosis factor-a, and IL-6 and glial fibrillary acidic protein in brain (14). We found that maternal methyl donor supplementation to cafeteria diet (CAF+M) normalized the sociability or social interaction in the 3-chamber test but not the non-social interaction in male offspring. However, we did not find that the control diet + methyl donor supplementation had a regulatory effect in sociability in offspring and we propose that the supplementation period—dose is key to establish beneficial epimutations (53) and in this case, the maternal CAF + methyl donor diet had a compensatory effect in the presence of this obesogenic stimuli.

The maternal CAF also promoted an anxiety-like behavior in offspring resulting from the time spent in the central and periphery zone in the open field test. In contrast with these results, Speight et al. found that a maternal CAF diet did not induce anxiety-like behavior in offspring in the open field test. They reported that these animals spent more time in the center zone and had more entries into this zone, also travelled a higher distance compared to the control group (54). Wright et al. established that a maternal CAF diet during the pre-gestational, gestational, and lactation period exerts an anxiolytic effect in male offspring shown by the presence of increased entries in the open arms in an elevated plus-maze and shorter latency to enter the center zone in open field test (55). Also, as described above, 5-HT is a monoaminergic neurotransmitter with activities that modulate central and peripheral functions. A recent study has shown that brain 5-HT deficiency can impair antidepressant responses and impact susceptibility to depression and anxiety-like behavior following stress (56). From this study, it was suggested that a maternal CAF diet promotes programming that could be regulating an anxiety-like behavior in the open field test. However, we observed that maternal methyl donor supplementation contributes to the presence of anxiety-like behaviors such as rearing and wall leaning, exploratory behaviors that have been also used to study anxiety-like behavior (57). Barua et al. reported that maternal folic acid supplementation leads to an increase in ultrasonic vocalizations and hyperactivity in offspring and that this could be mediated by a modification in the expression of genes implicated in releasing neurotransmitters and synaptic plasticity (58). Based on the above, it is likely that there is some epigenetic regulation at the brain level that could be associated with both the CAF diet and methyl donors. In this sense, mechanisms involved in brain development and maturation like the decreased proliferation of neuronal progenitor cells, abnormal synaptic stability sensitive to epigenetic regulation machinery, such as DNA methylation, may promote the development of alteration in the behavior of offspring born to obese mothers (59). Several nutrients included in these vitamins have a role in DNA and RNA synthesis and DNA methylation as well as for physiological and brain development (60). Methyl donors are key epigenetic mechanism regulators that can also be a component of the diet. In the brain, methyl group donors are important for multiple neurotransmitter pathways such as the acetylcholine synthesis, involved in memory and attention, with choline as a precursor (61).

Another mechanism involved can be due to altered levels of cortisol in offspring from dams supplemented with methyl donors (62) or due to a modulation in intestinal microbiota (63). These diets likely exert a decrease in the expression of DNMT (DNA methyltransferase) in these regions and, consequently, an alteration in behavior. Besides, the dietary pattern in industrialized countries has changed substantially over the past century due to technological advances in agriculture, food processing, storage, marketing, and distribution practices. In addition to being a predictor of obesity and metabolic dysfunction, consumption of a Western diet (WD) is related to poorer cognitive performance across the lifespan (64). Indeed, to the effect of perinatal WD exposure on anxiety-like behavior, studies suggest that maternal WD consumption throughout gestation and lactation may impact the HPC (hippocampus) and amygdala, brain regions that are strongly linked with anxiety. WD-associated anxiety-like behavior is accompanied by increased expression of 5HT-r1a and GABAa alpha2 receptor subunit in the ventral HPC (65), as well as elevated brain-derived neurotrophic factor (BDNF) expression in the dorsal HPC (66), a region where BDNF levels correlate with the magnitude of anxiety-like behavior in the elevated plus maze (EPM) task in wildtype mice (67). Exploration of the open arm of the EPM apparatus (decreased by WD) also correlates negatively with HPC gene expression for inflammatory markers TNFa and MCP-1 (68).

In the current study, we did not find any significant difference between groups in the marble burying test, a repetitive behavior test. This may be caused by heterogeneity in behavioral phenotype expressed by maternal diet or by a defensive-neophobic burying (69) or motor alterations that are regulated by an aberrant synaptic transmission of the cerebellar cortex (70). Indeed, in a recent study, the authors found an alteration in anxiety and impulsive behaviors for the HSB (high-sugar and fat)-fed mice through the marble burying test (71). Another study showed male-specific disturbances in social interaction and an increase in repetitive behavior during adolescence (72). Although there is not much evidence on CAF diet promoting a repetitive behavior, models such as valproic acid (22) have significant changes in the development of this behavior. Hill et al. found that a prenatal exposure to valproic acid promoted a decreased marble burying in offspring (73).

Otherwise, the amount and type of dietary macronutrients strongly influence the intestinal microbiota (74). Indeed, some individuals with neurodevelopmental disorders including ASD co-present with gastrointestinal problems and dysbiosis of the gut microbiota (75– 77). It has been shown that MHFD-induced obesity in mice is associated with social behavioral deficits, which are mediated by alterations in the offspring gut microbiome. Moreover, high-fat diet offsprings had shorter duration of social interaction and social contacts, in addition to showing no preference for social novelty in the 3-chamber sociability test. One of the factors involved in a marked social deficit in this group is the microbiota, the authors detected a reduced diversity and a difference in gut microbial communities compared to the control group (44). The intestinal microbiota may also be a factor that could be modulating anxiety-like behavior, as has been found in the study by Miousse et al. where methionine supplementation altered the composition of the microbiota (53).

## Conclusion

In conclusion, the maternal CAF diet promoted a decreased body weight and food intake in the offspring compared to the control diet group, suggesting that this diet did not affect programming an obesogenic phenotype in early life, although this diet caused an increase in EE that could develop a weight gain in later life by impairing basal metabolism. The CAF diet also contributed to a social deficit and anxiety-like behavior in the offspring. On the other hand, a maternal methyl donor-supplemented CAF diet normalized the social interaction in the offspring, although it led to an increase in anxiety-like behaviors. These findings suggest that a methyl donor supplementation could protect against aberrant social behavior probably targeting key genes related to neurotransmitter pathways.
